# Multiple Sclerosis Increases Fracture Risk: A Meta-Analysis

**DOI:** 10.1155/2015/650138

**Published:** 2015-02-01

**Authors:** Guixian Dong, Ning Zhang, Zhanpo Wu, Yumin Liu, Litao Wang

**Affiliations:** Department of Orthopedics, Harrison International Peace Hospital, Hengshui, Hebei 053000, China

## Abstract

*Purpose.* The association between multiple sclerosis (MS) and fracture risk has been reported, but results of previous studies remain controversial and ambiguous. To assess the association between MS and fracture risk, a meta-analysis was performed. *Method.* Based on comprehensive searches of the PubMed, Embase, and Web of Science, we identified outcome data from all articles estimating the association between MS and fracture risk. The pooled risk ratios (RRs) with 95% confidence intervals (CIs) were calculated. *Results.* A significant association between MS and fracture risk was found. This result remained statistically significant when the adjusted RRs were combined. Subgroup analysis stratified by the site of fracture suggested significant associations between MS and tibia fracture risk, femur fracture risk, hip fracture risk, pelvis fracture risk, vertebrae fracture risk, and humerus fracture risk. In the subgroup analysis by gender, female MS patients had increased fracture risk. When stratified by history of drug use, use of antidepressants, hypnotics/anxiolytics, anticonvulsants, and glucocorticoids increased the risk of fracture risk in MS patients. *Conclusions.* This meta-analysis demonstrated that MS was significantly associated with fracture risk.

## 1. Introduction

Multiple sclerosis (MS) is a chronic inflammatory demyelinating disease of the central nervous system (CNS) affecting primarily young adults [1]. MS is associated with an increased risk of osteoporosis and reduced bone mass [2, 3]. For example, in a relatively young (mean age: 51.29 ± 8.7 years) male MS population, 80% of cases could be defined as osteopenia or osteoporosis [4]. A study using North American Research Committee on Multiple Sclerosis (NARCOMS) Registry data reported that more than 25% of participants had low bone mass, while 15% of subjects had a history of fracture after age of 13 years [5]. Therefore, MS patients may have an increased risk of fracture.

Recently, several studies suggested that MS was associated with fracture risk [6–14]. However, the results were inconsistent. This meta-analysis aimed to determine the association between MS and fracture risk.

## 2. Methods

### 2.1. Publication Search and Inclusion Criteria

We searched the electronic databases PubMed, Embase, and Web of Science using the following search terms: (multiple sclerosis or MS) and (“fractures, bone” or fractures or fracture or “bone fractures”) without restriction on language. The included articles were published before June 2014. All eligible studies were retrieved, and their references were examined manually for other potentially relevant studies. The inclusion criteria were as follows: (1) cohort design or case-control design; (2) the association of MS with risk of fracture that should be evaluated.

### 2.2. Data Extraction

All data were independently collected from the included studies according to a standardized protocol by two investigators. The discrepancies during data extraction were resolved by consensus. The same data in different studies were used only once. The following information was extracted: first author's name, publication year, study design, age, gender, follow-up years, sample size, and covariants.

### 2.3. Statistical Analysis

The association between MS and fracture risk was assessed using risk ratio (RR) with 95% confidence interval (CI). The *Z* test was used to assess the pooled RR with the significance set at *P* < 0.05. The presence of between-study heterogeneity was evaluated by using the *I* ^2^ statistic test, which does not inherently depend on the number of studies in the meta-analysis and is preferable to the test of heterogeneity. The value of *I* ^2^ ranged from 0 to 100%. If obvious heterogeneity was observed among the studies (*I* ^2^ > 50%), the random-effects model (the DerSimonian and Laird method) was used to calculate the pooled RR and 95% CI. Otherwise, the fixed-effects model (the Mantel-Haenszel method) was adopted for the meta-analysis. Subgroup analyses according to the site of fracture, gender, and history of drug use were also performed. We carried out the cumulative meta-analysis. Sensitivity analyses were conducted to evaluate the effect of individual study on pooled results and assess the stability of results. The potential publication bias was detected with Begg's funnel plot, and the funnel plot asymmetry was assessed by Egger's linear regression test [15]. All statistical analyses were performed using the STATA 12.0 software (StataCorp, College Station, TX, USA).

## 3. Results

### 3.1. Characteristics of Eligible Studies

A total of 9 cohort studies with 9,229,368 subjects met the inclusion criteria [6–14]. The mean age ranged from 37 years to 65 years. Most of the included subjects were females. The follow-up ranged from 3 years to 20 years. Data collected from the included studies were summarized in Table 1.

### 3.2. Results of Meta-Analysis

**Figure 1:**
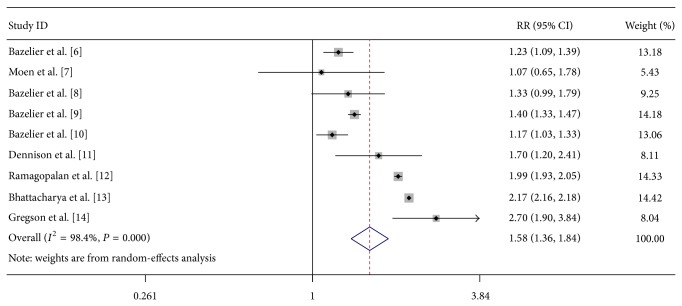
Forest plot of the overall risk of fracture associated with MS.

As shown in Figure 1, a significant association between MS and fracture risk was found (RR = 1.58, 95% CI 1.36–1.84, *P* < 0.00001). This result remained statistically significant when the adjusted RRs were combined (RR = 1.62, 95% CI 1.17–2.24, *P* = 0.004). Subgroup analysis stratified by the site of fracture suggested significant associations between MS and tibia fracture risk (RR = 2.87, 95% CI 2.35–3.52, *P* < 0.00001), femur fracture risk (RR = 4.87, 95% CI 3.39–6.99, *P* < 0.00001), hip fracture risk (RR = 3.18, 95% CI 2.84–3.56, *P* < 0.00001), pelvis fracture risk (RR = 1.55, 95% CI 1.38–1.74, *P* < 0.00001), vertebrae fracture risk (RR = 1.44, 95% CI 1.16–1.78, *P* = 0.001), and humerus fracture risk (RR = 1.56, 95% CI 1.09–2.24, *P* = 0.02). However, there was no significant association between MS and ribs fracture risk (RR = 1.14, 95% CI 0.79–1.64, *P* = 0.48) and radius/ulna fracture risk (RR = 0.92, 95% CI 0.83–1.02, *P* = 0.13). In the subgroup analysis by gender, female MS patients had increased fracture risk (RR = 1.80, 95% CI 1.61–2.01, *P* < 0.00001). In contrast, no significant association was observed in the male MS patients (RR = 1.18, 95% CI 0.77–1.81, *P* = 0.45). When stratified by history of drug use, use of antidepressants, hypnotics/anxiolytics, anticonvulsants, and glucocorticoids increased the risk of fracture risk in MS patients (Table 2).

**Figure 2:**
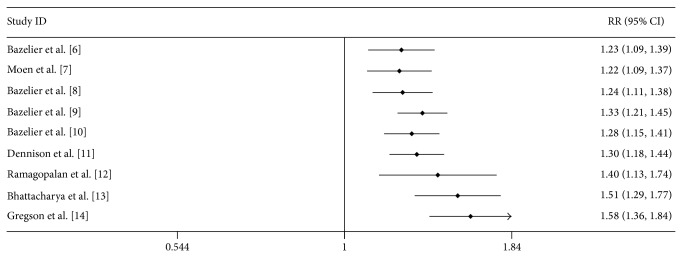
Cumulative meta-analysis of the association between MS and fracture risk.

**Figure 3:**
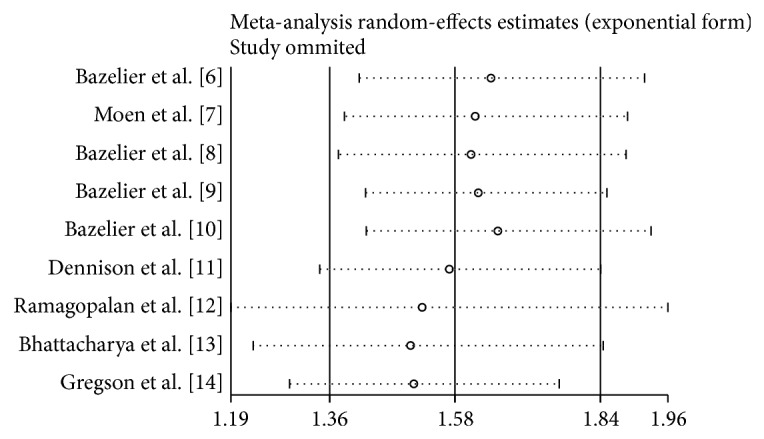
Sensitivity analysis of the association between MS and fracture risk.

As shown in Figure 2, cumulative meta-analysis showed that the pooled RRs tended to be stable. A single study was excluded each time to evaluate the effect of individual study on the combined RRs and 95% CIs. The omission of any single study did not make significant difference in the pooled effects of additive model, suggesting a high stability of meta-analysis results (Figure 3).

**Figure 4:**
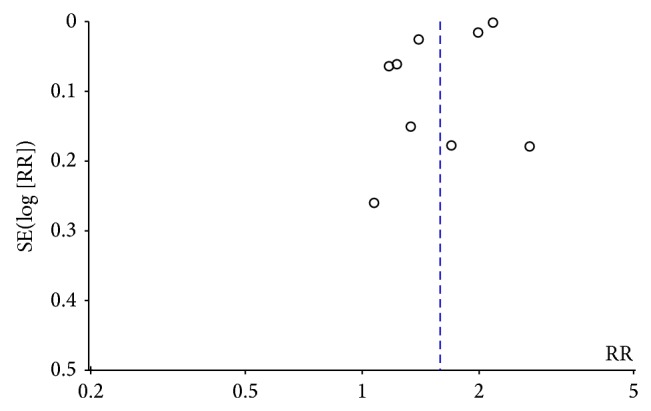
Funnel plot of the association between MS and fracture risk.

Publication bias of the selected articles was assessed by Begg's funnel plot and Egger's test. The shape of the funnel plot did not show obvious publication bias (Figure 4). Similarly, no evidence of publication bias was observed by Egger's test (*P* = 0.22).

## 4. Discussion

In the current meta-analysis with 9,229,368 subjects, we found that there was a significant association between MS and fracture risk, even after excluding the studies without adjusting covariants. Further stratified analysis revealed that MS was significantly associated with fracture risk in female patients but not in male patients. This may be explained by lower bone mass in women with MS [5]. After stratification by site of fracture, this association remained significant in tibia, femur, hip, pelvis, vertebrae, and humerus. Although the result was not statistically significant in ribs and radius/ulna, the positive association could not be ruled out because studies with small sample size may have insufficient statistical power to detect a slight effect. More studies are needed to address the association between MS and ribs or radius/ulna fracture risk. In addition, among users of antidepressants, hypnotics/anxiolytics, anticonvulsants, and glucocorticoids, we found significantly more fractures associated with MS. These results indicated that MS patients who received these drugs should be paid more attention.

There were several possible reasons to explain the finds of this meta-analysis. First, MS patients usually had decreased bone mineral density (BMD). Dobson et al. [16] found that the overall BMD was much lower in MS patients. Second, symptoms of MS include muscle weakness, poorer postural balance, stiffness, numbness, tingling, impaired vision, fatigue, dizziness, disability, or spasticity. Each symptom could exert a role in the etiology of falls that are very common in MS patients.

Between-study heterogeneity is common and should be explored in the meta-analysis. In the current study, significant heterogeneity was found in the association of MS with fracture risk. Therefore, subgroup analyses were performed to explore the sources of between-study heterogeneity. The results indicated that gender might be the source of heterogeneity. In the male and female subgroups, the heterogeneity was decreased significantly. Sensitivity analysis revealed that the omission of any single study did not have significant impact on the overall meta-analysis estimate. Furthermore, in the meta-analysis, funnel plot did not reflect considerable asymmetry and Egger's test also indicated no obvious publication bias. All these made the meta-analysis results reliable to some extent.

There are some limitations in this meta-analysis. First, the retrieved literature was potentially not comprehensive enough. We did not track the unpublished articles to obtain data for analysis. The potential effect of this publication bias was unknown. Second, fracture is a multifactorial disease and potential interactions among MS-environment should be considered. Moreover, as many other factors such as age may participate in the progression of fracture, we did not carry out subgroup analysis based on these factors due to limited data. Finally, the severity of MS could potentially be a confounder. Most studies included MS patients with mild to moderate severity, but more severe patients were the ones with walking issues and falling issues. However, we did not adjust the severity of MS in this meta-analysis. More studies should include the severity of MS, such as the Expanded Disability Status Scale (EDSS), as a confounder in the future.

In conclusion, a significant association was detected between MS and fracture. Moreover, further studies with large sample size will be necessary to validate this result.

## Figures and Tables

**Table 1:** Characteristics of the included studies.

First author	Year	Study design	Mean age	Women (%)	Follow-up years	Sample size	Adjusted for
Bazelier [6]	2011	Cohort	44.8	70	11	38925	Age, sex, the use of oral/intravenous glucocorticoids, antidepressants, hypnotics/anxiolytics, anticonvulsants, and opioids in the previous 6 months, history of falling at index date, history of fracture at index date, history of cerebrovascular disease, epilepsy, history of smoking, BMI

Moen [7]	2011	Cohort	37.1	72	NA	231	NA

Bazelier [8]	2012	Cohort	43.6	72	11	15056	Age, sex, the use of antidepressants, anticonvulsants, and bisphosphonates in the previous 6 months

Bazelier [9]	2012	Cohort	46.4	66	12	68430	NA

Bazelier [10]	2012	Cohort	36.9	67	7.2	18399	Age, sex, the use of oral/intravenous glucocorticoids, antidepressants, hypnotics/anxiolytics, anticonvulsants, and opioids in the previous 6 months, history of cerebrovascular disease, epilepsy

Dennison [11]	2012	Cohort	NA	100	3	52960	Heart disease, osteoarthritis, chronic obstructive pulmonary disease, Parkinson's disease

Ramagopalan [12]	2012	Cohort	NA	69	11	7908570	NA

Bhattacharya [13]	2014	Cohort	65	79	20	1066404	Sex, race

Gregson [14]	2014	Cohort	≥55	100	3	60393	Age

NA, not available.

**Table 2:** Results of this meta-analysis.

	Test of association			Model	Heterogeneity		
	RR (95% CI)	*Z*	*P* value		*χ* ^2^	*P* value	*I* ^2^ (%)
All studies	1.58 (1.36–1.84)	5.98	<0.00001	R	508	<0.0001	98
Adjusted	1.62 (1.17–2.24)	2.89	0.004	R	188.32	<0.0001	97
Site							
Tibia	2.87 (2.35–3.52)	10.27	<0.00001	R	5.55	0.06	64
Femur	4.87 (3.39–6.99)	8.59	<0.00001	R	7.86	0.02	75
Hip	3.18 (2.84–3.56)	20.08	<0.00001	F	1.08	0.78	0
Pelvis	1.55 (1.38–1.74)	7.39	<0.00001	F	0.53	0.77	0
Vertebrae	1.44 (1.16–1.78)	3.29	0.001	F	1.87	0.60	0
Ribs	1.14 (0.79–1.64)	0.71	0.48	R	8.38	0.04	64
Radius/ulna	0.92 (0.83–1.02)	1.51	0.13	F	1.85	0.40	0
Humerus	1.56 (1.09–2.24)	2.41	0.02	R	23.79	<0.0001	87
Gender							
Male	1.18 (0.77–1.81)	0.75	0.45	R	2.45	0.12	59
Female	1.80 (1.61–2.01)	10.32	<0.00001	F	0.18	0.67	0
History of drug use							
Antidepressants	1.95 (1.37–2.77)	3.70	0.0002	R	4.03	0.13	50
Hypnotics/anxiolytics	1.88 (1.09–3.23)	2.28	0.02	R	8.44	0.01	76
Anticonvulsants	1.80 (1.31–2.46)	3.67	0.0002	F	0.79	0.37	0
Glucocorticoids	1.33 (1.11–1.59)	3.05	0.002	F	0.44	0.80	0

RR, risk ratio; CI, confidence interval; R, random-effects model; F, fixed-effects model.
